# Shifts in soil microbial and nematode communities over progression of pine wilt disease occurring in *Pinus koraiensis* stands

**DOI:** 10.3389/fmicb.2025.1634289

**Published:** 2025-11-24

**Authors:** Siyu Tian, Mingwei Wang, Xin Dong, Yuting Ji, Hao Wu, Tuuli-Marjaana Koski, Minggang Wang, Qi Li

**Affiliations:** 1 The Key Laboratory for Silviculture and Conservation of Ministry of Education, College of Forestry, Beijing Forestry University, Beijing, China; 2 Liaoning Forestry and Grassland Bureau, Shenyang, China; 3 Liaoning Provincial Key Laboratory of Dangerous Forest Pest Management and Control, Shenyang Institute of Technology, Shenyang, China; 4 State Key Laboratory of Tree Breeding, College of Biological Sciences and Technology, Beijing Forestry University, Beijing, China; 5 Department of Biology, University of Turku, Turku, Finland; 6 Ecological Observation and Research Station of Heilongjiang Sanjiang Plain Wetlands, National Forestry and Grassland Administration, Shuangyashan, China; 7 Key Laboratory of Terrestrial Ecosystem Carbon Neutrality, Liaoning Province, Institute of Applied Ecology, Chinese Academy of Sciences, Shenyang, China

**Keywords:** disease development, pinewood nematode, soil nematode community, soil microbial community, *Pinus koraiensis*

## Abstract

**Introduction:**

Pine wilt disease (PWD) is recognized as a destructive forest disease worldwide, leading to massive mortality of many *Pinus* spp., including the Korean white pine *Pinus koraiensis*. Current work has focused on underlying development of this disease occurring aboveground, but few studies have assessed soil consequences from the destruction in pine forest by PWD.

**Methods:**

In this study, we collected soil samples from one stand of PWD-resistant species *Larix olgensis*, and from four stands of PWD-susceptible *P. koraiensis* (*n* = 8) following a natural chronosequence of PWD development (healthy, diseased, killed, and clear-cut *P. koraiensis*). We aimed to investigate the shifts in soil microbial and nematode communities under the canopy of *P. koraiensis* over the PWD progression.

**Results:**

The α-diversity e.g., species richness of bacterial community in soil of healthy P. koraiensis was ca. 17% lower than in soil of diseased pines. The species richness of fungal community in the soil of healthy P. koraiensis was also 24.5% lower than in soil of killed pines. The diseased and killed pines also exhibited different compositions in soil microbial community from the healthy pines, although these damaged trees did not differ themselves in the composition. In particular, the relative abundance of the methane-cycling Methylomirabilota became higher in bacterial community and the ectomycorrhizal Agaricomycetes was lower in fungal community in soil of the diseased or killed pines than healthy ones, suggesting an overall decrease in soil health caused by PWD. Although the α-diversity of soil nematode community did not vary over the development of PWD, its composition was significantly altered by the disease. Consequently, we observed a lower inter-kingdom network complexity in the soil community of the pines following the PWD, in which the bacterial networks decreased but fungal networks increased in complexity. The nematode community also showed a lower network complexity in soil of PWD-destructed pines, albeit that this only occurred when the pines were diseased rather than killed.

**Discussion:**

By recording the structure dynamics of soil microbial and nematode communities in pines following the progression of PWD, this study helps to understand the impacts of PWD on soil biotic processes, thus providing an important reference for better assessing the ecological consequences of this devastating disease.

## 1 Introduction

Pine Wilt Disease (PWD) is a destructive disease of pine forests that originates from North America but has invaded large regions of Asia and Europe. This disease has killed millions of pine trees (*Pinus* spp.) and resulted in substantial economic and ecological losses in its invasive area (Xu et al., 2023; Chen et al., 2024). PWD arises from a complex interplay among the host pine, the pathogenic agent pinewood nematode (PWN, *Bursaphelenchus xylophilus*), and the vector long-horned beetle (*Monochamus* spp., Cerambycidae) as well as the associated microbes (Steiner and Buhrer, 1934; Evans et al., 1996; Dou and Yan, 2022; Koski et al., 2024). The infection occurs when the long-horned beetles carry PWN in their digestive systems and attack a healthy pine host. During the feeding on pine twigs, the nematodes migrate from the beetle's mouthparts into the tree's vascular system (Liu et al., 2021). Once inside, the nematodes rapidly reproduce and cause tissue disintegration and blocking water-conducting vessels, which often leads to tree decline and death even in a few weeks (Zhao et al., 2008).

Korean white pine (*Pinus koraiensis*) is an economically and ecologically valuable pine species that widely distributes in Northeast China and has experienced severe destruction by PWD in recent years (Yu and Wu, 2018; Pan et al., 2019). The species *P. koraiensis* is distributed across Northeast China, Southeast Siberia, and the northern part of Korea, with additional populations occurring on Honshu and Shikoku islands in Japan (Zhao et al., 1991). This species was recently confirmed as a natural host of PWD through infection records (Yu and Wu, 2018; Pan et al., 2019). In 2017, an extensive dieback of *P. koraiensis* occurred in our experimental area, which initiated a field survey that eventually confirmed for the first time *P. koraiensis* as a natural host of pinewood nematodes vectored by the long-horned pine sawyer beetle, *Monochamus saltuarius* Gebler (Yu and Wu, 2018; Hou et al., 2021). The infection of *P. koraiensis* by PWN typically begins in July, and evident symptoms can be observed on average 81 days after the infestation (Pan et al., 2019). On the other hand, another coniferous species, *L. olgensis* from the family Pinaceae, which co-occurs in our experimental site was recognized to be resistant to the PWD (Fang et al., 2022; Jiang et al., 2022; Wang et al., 2024). Therefore, *Larix* spp. are often considered valuable for studying resistance-related traits of pines against PWD (Wang et al., 2024).

Soil biota, in particular the microbiota associated to plant roots, are important to plant growth and health (Wei et al., 2019; Pineda et al., 2020; van Dijk et al., 2022). The root-associated soil microbes sustain plant performance through their fundamental roles in nutrient provisioning and stress-resistance induction (Trivedi et al., 2020; Das et al., 2022). The roles of these soil microbes are also related to community dynamics of microbial or plant consumers (e.g. soil animals) in soil, consisting of soil food webs with plant roots or rhizodeposits via trophic networks and contributing to various ecosystem functions (Kou et al., 2023; Shi et al., 2024). Soil nematodes are the most abundant soil animals that can intimately interact with plants (Wilschut and Geisen, 2021). Plant-feeding nematodes often directly harm the plants by parasitizing their hosts while the microbial-feeding nematodes may indirectly facilitate the plants via trophic cascade that mineralize the microbial-assimilated nutrients for plant use (Potapov, 2022; Li G. et al., 2024). Many studies have shown the dynamics of microbial communities in different compartments of the host pines, including the communities in soil following the natural occurrence of PWD (Hao et al., 2022; Guo et al., 2023; Hou et al., 2025). However, to our knowledge, the responses of soil animals e.g., soil nematodes that consume these microbes to PWD have not yet been investigated. Particularly, whether and to what extent the two soil groups differ in their responses to PWD remains unknown.

In the current study, we sampled soils under canopy of Korean white pines suffering different stages of PWD to examine the responses of associated bacterial, fungal, and nematode communities of this pine species to the progression of PWD. We hypothesize that (1) structures of both soil microbial and nematode communities are influenced by PWD, (2) but the influences differ between microbial and nematode communities, and (3) these influences become stronger over the progression of the PWD.

## 2 Materials and methods

### 2.1 Study site

The study was conducted at Dahuofang Forest Station in Fushun City, Liaoning Province, Northeast China (124°16′E, 41°58′N). The dominant tree species are *P. koraiensis, Pinus tabuliformis* (Chinese pine), and *Larix olgensis* (Changbai larch) in the forest station (Yu et al., 2015). This region was found natural occurrence of PWD in 2017 and recorded as the northernmost boundary of the PWD epidemic area in China (Yu and Wu, 2018). It has a typical continental monsoon climate within the mid-temperate zone. The mean annual temperature in this region is 5.5 °C, and the mean annual precipitation is 661.22 mm as well as mean annual evaporation is 1,411.78 mm according to CGIAR-CSI Global Aridity and PET Database (Zomer et al., 2022). The pine forests of *P. koraiensis* were established in the 1960s as pure stands, and all the pine individuals in the forests were similar in age (ca. 60 years old) and size (Hou et al., 2025).

### 2.2 Experimental design

**Figure 1:**
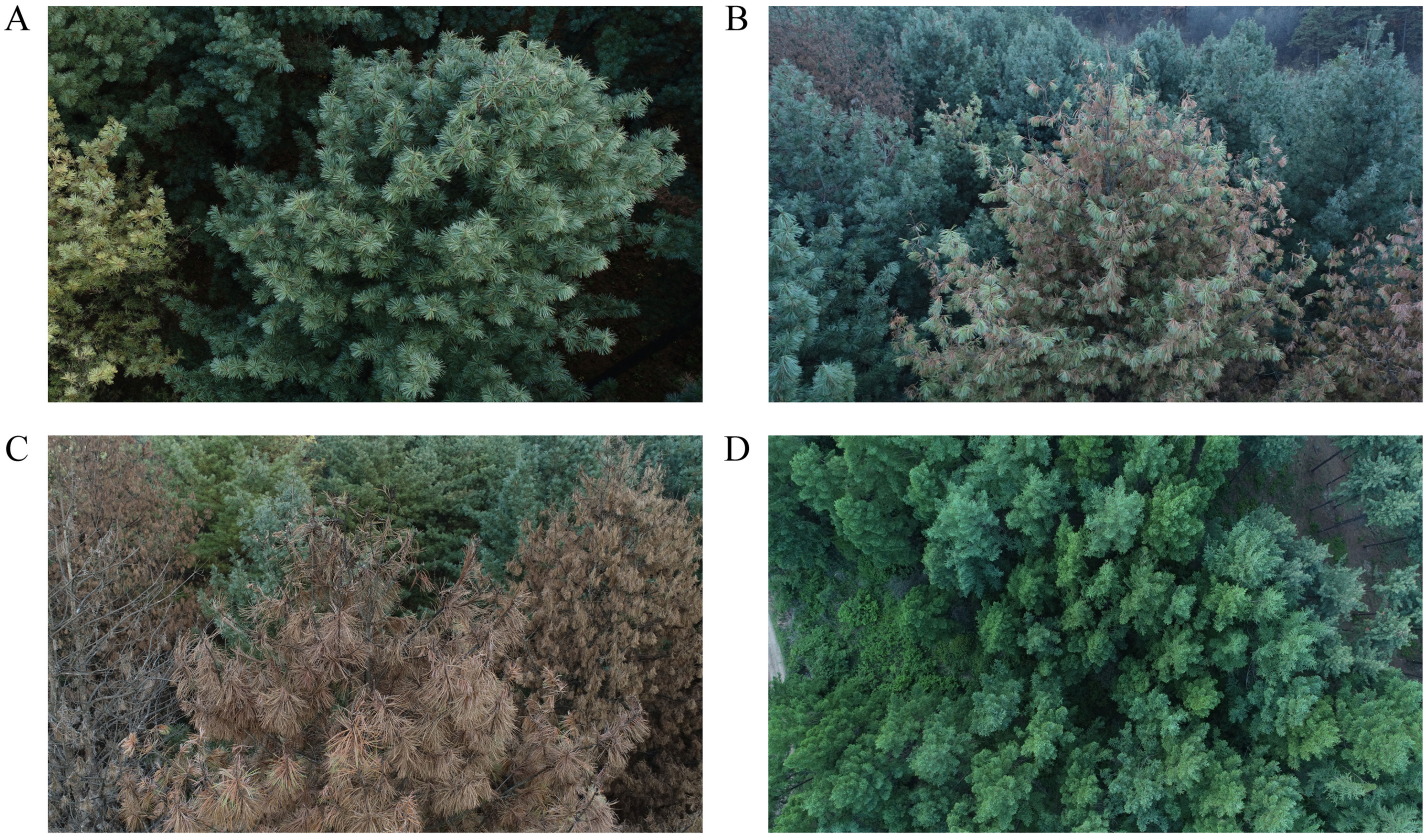
Overview of the susceptible Korean white pine *Pinus koraiensis* that were healthy **(A)**, diseased **(B)**, and killed **(C)** by PWD, as well as larch species *Larix olgensis* resistant to PWD **(D)** in the experimental site.

The study was conducted in early October 2021 when the symptoms of newly infected pines with PWD could be visually determined (Ma et al., 2020; Hou et al., 2025). In the forest station, we selected four forest stands of *P. koraiensis* that follow a natural chronosequence of PWD, and a healthy *L. olgensis* stand. The selection was made based on unmanned aerial vehicle remote sensing data of discolored standing trees in the experimental site (Figure 1). In the stand of *P. koraiensis* where all the needles of each individual tree were reddish-brown, the trees were classified as fully killed by PWD (abbreviated as Killed *Pk*). In the neighboring stands where the pines had the majority needles of mixed colors of red and green, and these pines were considered diseased due to PWD (abbreviated as Diseased *Pk*). The healthy stand of *P. koraiensis* was composed of pine trees that had entirely green needles (abbreviated as Healthy *Pk*). A piece of triangle-shaped tissue was taken from the trunk of sampled individuals in each stand at breast height using a chain saw. The tissues were immediately transported to the laboratory and extracted using the “Baerman funnel method”. The microscopic examination verified the absence of *B. xylophilus* in the tissue of healthy pines and presence of *B. xylophilus* in the tissue of diseased and killed pines. In order to compare the soil communities of resistant vs. susceptible conifer species, we also selected a stand of larch species *L. olgensis* (abbreviated as Healthy *Lo*) in the vicinity of the killed stands of *P. koraiensis* (Fang et al., 2022; Wang et al., 2024). In addition, we also selected an open field nearby that had been created by clear-cutting the killed *P. koraiensis* (abbreviated as Clear-cut *Pk*) in the previous year. The inclusion of this field in the design was used to understand the consequences of PWD for soil microbial and nematode communities. It is noted that the ideal design was to select multiple stands that experienced similar levels of disease to avoid the pseudoreplication of sampling in the study. However, since the infection of *P. koraiensis* by PWD only recently occurred and the epidemic area was very small, such design was very difficult in practice. Therefore, we selected these forest stands that were spaced a minimum of 5 km in distance to minimize the effects of pseudoreplication on sampling errors. This selection was also to prevent the natural infection between the stands by the PWD at the time of sampling, given that the maximal spread distance of the vectored beetles was 5 km (Hou et al., 2025).

Within each forest stand, eight individuals of *P. koraiensis* or *L. olgensis* were randomly selected and the individuals were at least 30 m in distance between each other) and the surface soil under their canopy was collected (see below for details). Therefore, a total of 40 soil samples were collected (5 sampling fields × 8 samples per field) in the current study. All experimental sites were located on a hilly terrain orienting south to north, with slopes ranging from 40° to 50°. The sampled soils are of dark brown forest soils that have averaged pH 6.59, contents of 5.21 g·kg−1 total carbon, 0.38 g·kg−1 total nitrogen, and 0.78 g·kg−1 total phosphorus (Hou et al., 2025).

### 2.3 Soil sampling

Before sampling, the understory vegetation and litter around each tagged tree were removed using a sterilized shovel. Topsoil at a 0–15 cm depth was collected from each of the four directions ca. 45 cm from the trunk using a soil sampler (38 mm in diameter). The four soil cores from each individual tree were thoroughly mixed to form a composite soil sample and placed in a new plastic bag. All the collected samples were stored in an ice box and immediately transported to the laboratory at the Shenyang Institute of Technology, Shenyang, China. The shovel and soil corer were sterilized using 75% alcohol between sampling events. In the laboratory, each soil sample was divided into three subsamples for microbial DNA extraction, nematode extraction, and physicochemical analyses.

### 2.4 Soil nematode extraction and identification

Soil nematodes were extracted from 100 g fresh soil sample using a modified Baermann funnel method (Viglierchio and Schmitt, 1983). The extracted soil nematodes were preserved in formalin for later identification. The first 100 encountered nematodes in each sample were identified using a microscope (OLYMPUS CKX53, Tokyo, Japan). If the sample contained fewer than 100 nematodes, all the nematodes were identified. Nematodes were determined at the genus level according to identification guides of Bongers (1988) and Zhang et al. (2013). The nematode density was calculated to be total number of individuals per 100 g dry soil. All nematodes were further categorized into plant feeders (PF), bacterial feeders (BF), fungal feeders (FF), carnivores (CA), and omnivores (OM) according to their feeding habits (Yeates et al., 1993).

### 2.5 Soil physicochemical properties

All samples were analyzed for pH, soil water content (SWC), total carbon (TC), total nitrogen (TN), and total phosphorus (TP). Soil pH was determined at a soil-to-water ratio of 1:2.5 (w/v) by the pH meter S210–K (Mettler Toledo, Germany). SWC was estimated by oven-drying the samples at 105 °C to constant weight. TC and TN were analyzed using a CN elemental analyzer (Elementar Vario EL III, Elementar, Germany). TP was measured by colorimetric analysis after digestion with sulfuric acid and perchloric acid.

### 2.6 DNA extraction and quantitative PCR

Soil microbial genomic DNA was extracted using the PowerSoil DNA Isolation Kit (Omega Bio-tek, Norcross, GA, USA) following the manufacturer's protocol. The primers 338F (5′-ACTCCTACGGGAGGCAGCAG-3′)/806R (5′-GGACTACHVGGGTWTCTAAT-3′) were used to amplify V3-V4 regions of the bacterial 16S rRNA gene and the primers ITS1F (5′-CTTGGTCATTTAGAGGAAGTAA-3′)/ITS2R (5′-GCTGCGTTCTTCATCGATGC-3′) were used to amplify the fungal ITS1 region (Crowther et al., 2014). Genomic DNA from each sample was independently amplified and the PCR products were pooled and purified using the QIAquick Gel Extraction Kit (Qiagen). Subsequently, the concentration of purified amplicons was quantified using the Picogreen assay (Invitrogen) on a TBS-380 microplate reader, and equimolar concentrations were mixed. Finally, the pooled samples were subjected to paired-end sequencing (2 × 250 bp) on the Illumina Novaseq 6000 platform at Majorbio Bio-Pharm Technology Co., Ltd. (Shanghai, China). One bacterial sample from the clear-cut soil stand was excluded due to failed amplification.

### 2.7 Bioinformatics of sequencing data

The initial sequence processing was conducted using QIIME2 (version 2021.2) (Bokulich et al., 2018). Samples were first demultiplexed using the Demux plugin, followed by adapter and Barcode sequence removal using the Cutadapt plugin (Martin, 2011). Subsequently, the DADA2 plugin was employed for filtering, denoising, and quality control, resulting in the generation of the Amplicon Sequence Variants (ASV) table (Callahan et al., 2016). For bacterial sequences, taxonomic classification was performed using the SILVA (V138.1) database (Quast et al., 2013). For fungal sequences, the UNITE (V8.2) database (Kõljalg et al., 2013) was used for species annotation of ASV sequences. Finally, data were rarefied across samples for subsequent biodiversity analysis and community structure comparisons.

### 2.8 Prediction for functions of microbial taxa

FAPROTAX was used to predict bacterial ecological functions by analyzing gene sequences and inferring their functional potential, such as metabolic functions and aromatic compound degradation (Louca et al., 2016; Sansupa et al., 2021). For the fungal data, we used FUNGuild to assign fungal sequencing data into three trophic groups (symbiotroph, saprotroph, and pathotroph), by comparing them against a database of known fungal genes associated with specific nutritional modes (Nguyen et al., 2016). Sequences that were assigned more than one trophic strategy in FUNGuild were excluded from our analysis (Deng et al., 2023). For each soil sample, the relative abundances were calculated for each functional or trophic group.

### 2.9 Analysis of co-occurrence networks in microbial and nematode communities

To explore the co-occurrence patterns of bacterial, fungal, and nematode communities in different forest types, correlation-based network analyses were conducted for each community as well as inter-kingdom network analysis. Only bacterial ASVs, fungal ASVs, and nematode genera present in at least 25% of the samples were included to ensure correlation reliability. To select highly significant associations, overall networks were constructed using correlations that were significant (*p* < 0.05) with strong positive (*r* > 0.6) or strong negative (*r* < −0.6) coefficients. Additionally, to simplify networks for better visualization and analysis, we used ASVs with relative abundance higher than 0.01% to construct the microbial networks for different soil origins (Khatri-Chhetri et al., 2024). Relationships among microbial and nematode communities were visualized in Gephi (version 10.1) (Bastian et al., 2009) using the Fruchterman Reingold layout (Fruchterman and Reingold, 1991). Various network structural attributes including node level topological properties such as degree and density were calculated in package “igraph”. Nodes (ASVs of bacteria and fungi, and genera of nematodes) are the essential units of a network, while edges represent links between nodes.

### 2.10 Data analysis

The α-diversity indices including species richness and Shannon index (*H'*), as well as β-diversity were calculated using the “vegan” package (Oksanen et al., 2013). We employed one-way ANOVA to examine the effects of PWD chronosequence on diversities of soil microbial and nematode communities, in which α-diversity indices were included as response variables and “soil origin” as a fixed factor. Tukey's HSD (Honest Significant Difference) *post hoc* tests were used to compare α-diversity among soil origins. Principal coordinate analysis (PCoA) was used to visualize the separations of the communities. Effects of PWD on soil microbial and nematode community composition were assessed by permutation multivariate analysis of variance (PERMANOVA) with Bray-Curtis dissimilarity (999 permutations) in “vegan” package (Oksanen et al., 2013). Canonical Correspondence Analysis (CCA) was performed to examine the relationships between soil biota and soil physiochemical properties. Prior to the CCA, the Detrended Canonical Analysis (DCA, 999 permutations) was performed to assess the explained variance of each environmental factor, using the “envfit” function in “vegan” package (Dong et al., 2024). Differential relative abundances of taxa and functions were analyzed using the Kruskal-Wallis test with Benjamini-Hochberg correction in Statistical analysis of taxonomic and functional profiles (STAMP) software (Parks et al., 2014). Kruskal-Wallis test was alternatively used in the “agricolae” package when the assumptions were not met even after data transformation. The *p*-values of the Kruskal-Wallis test were adjusted using the Benjamini-Hochberg method to control the false discovery rate (FDR). All statistical analyses and data visualizations were performed using R version 4.4.1 (R Core Team, 2024).

## 3 Results

In total, there were 10,625 bacterial ASVs and 9,565 fungal ASVs detected from all the 40 soil samples. Among them, 791 bacterial ASVs (7% out of the total number of ASVs) and 116 fungal ASVs (1% out of the total number of ASVs) were shared by all five treatments (Supplementary Figures 1A, B). A total of 68 nematode genera were identified, and 30 nematode genera (44% out of the total number) were shared by all the treatments (Supplementary Figure 1C).

### 3.1 The α-diversity of microbial and nematode community

**Figure 2:**
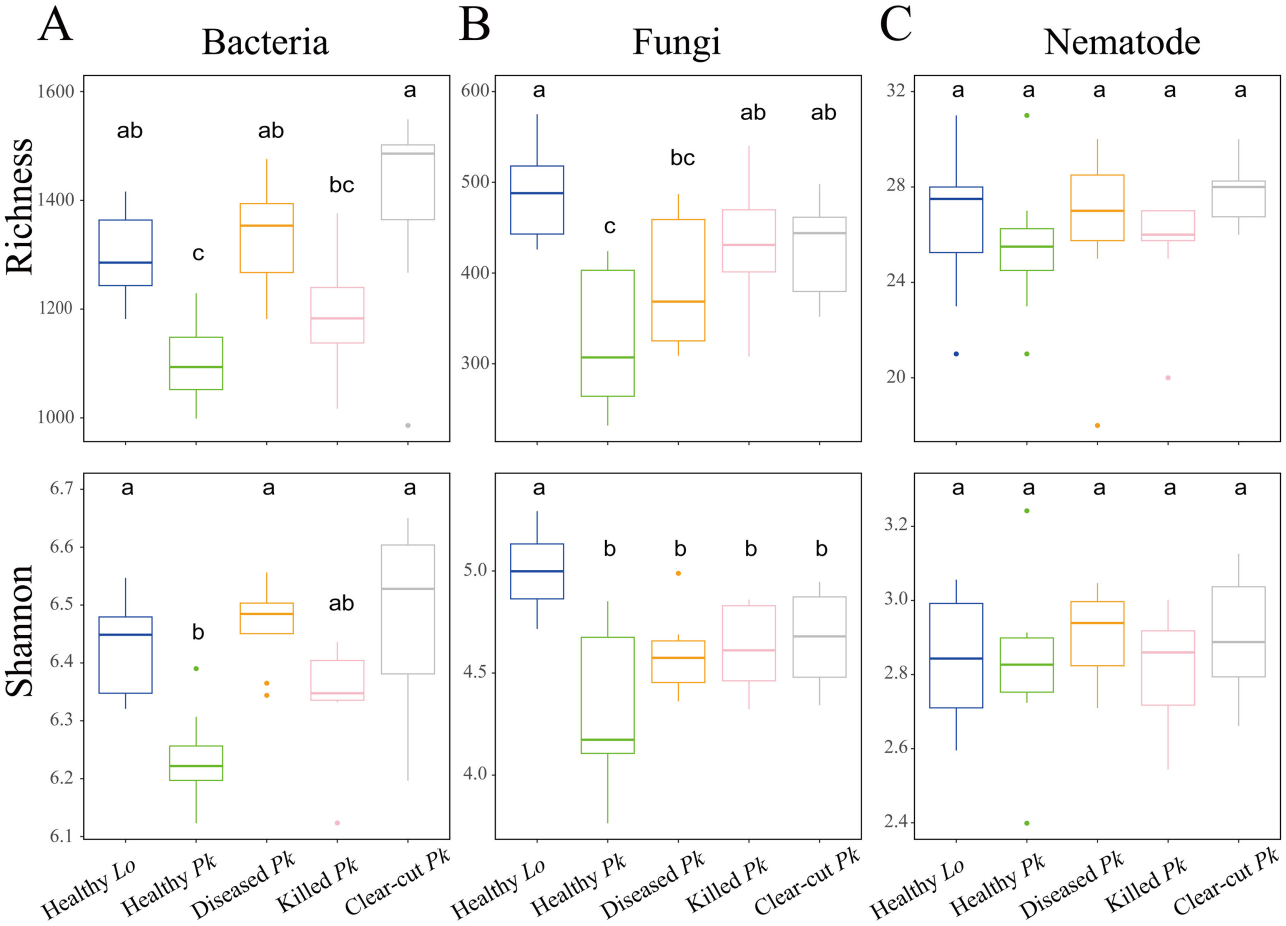
Boxplots showing the differences in α-diversity indices (Shannon and Richness) of bacterial **(A)**, fungal **(B)**, and nematode **(C)** communities in soils collected from healthy larch *L. olgensis* stand resistant to PWD, healthy *P. koraiensis* stands, diseased and killed *P. koraiensis* stands by PWD as well as clear-cut *P. koraiensis* stands. The boxplot shows the 25% and 75% quartiles, the median, whiskers (1.5 times the interquartile range) and the outliers of the samples. Shared letters are not significantly different from each other based on Tukey's *post hoc* test (Tukey's HSD, *p* > 0.05).

The species richness of bacterial community in soil of healthy *P. koraiensis* was 20.8% lower than in clear-cut soil and 17.3% lower than in soil of diseased pines, respectively (Figure 2A). Similarly, the species richness of fungal community in soil of healthy *P. koraiensis* was lower than in clear-cut soil (by 24.1%) and in soil of killed pines (by 24.5%) (Figure 2B). The Shannon index of both bacterial and fungal communities was also significantly lower in the soil of healthy pines than in soils of other origins including in soil of larch, but the differences were relatively small (all <5.0%) (Figures 2A, B). However, this difference in bacterial diversity disappeared when *P. koraiensis* was killed by PWD (Figure 2A). On the contrary, the diversity of soil nematode community did not differ across different soil origins (Figure 2C).

### 3.2 The composition of soil microbial and nematode community

**Figure 3:**
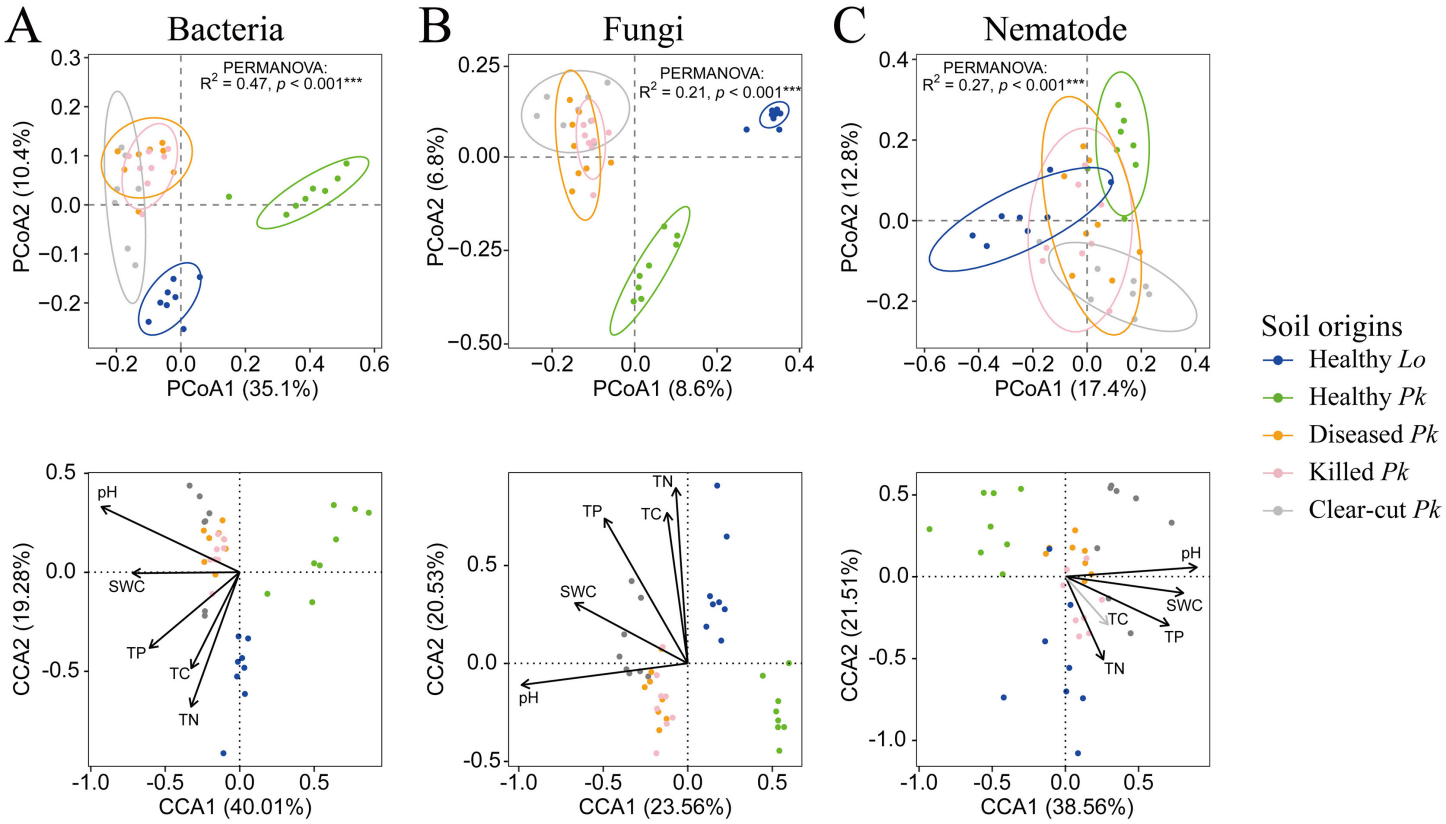
Ordination diagram of principal coordinates analysis (PCoA) of bacterial **(A)**, fungal **(B)**, and nematode **(C)** communities in soils collected from healthy larch *L. olgensis* stand resistant to PWD, healthy *P. koraiensis* stands, *P. koraiensis* stands diseased by PWD, and *P. koraiensis* stands killed by PWD as well as clear-cut *P. koraiensis* stands. Variances in the data explained by soil origins were tested with PERMANOVA based on Bray-Curtis dissimilarity. Canonical correspondence analysis (CCA) was performed to identify soil parameters that potentially influence the composition of bacterial, fungal, and nematode community. The results are presented alongside the PCoA ordinations. Abbreviations for soil origins: SWC, soil water content; TC, total carbon, TN, total nitrogen; TP, total phosphorus.

The first two axes of PCoA explained 35.1% and 10.4% of the variance in soil bacterial community, respectively. The composition of bacterial community in the soil of healthy *P. koraiensis* differed from that in soil of larch, as well as from the compositions in soil of diseased or killed *P. koraiensis* trees, although the latter two soils did not differ (PERMANOVA: R^2^ = 0.47, *p* < 0.001; Figure 3A and Supplementary Table 1). A similar pattern was observed for both soil fungal community (8.6% and 6.8% for PC1 and PC2, respectively; PERMANOVA: R^2^ = 0.21, *p* < 0.001; Figure 3B and Supplementary Table 1) and nematode community (17.4% and 12.8% for PC1 and PC2, respectively; PERMANOVA: R^2^ = 0.27, *p* < 0.001; Figure 3C and Supplementary Table 1). The results of CCA revealed that edaphic factors including pH, SWC, TC, TN, and TP all significantly contributed to the difference in the compositions of microbial and nematode communities of these soil origins, except TC that was not related to the composition of nematode communities (Figure 3 and Supplementary Table 2). However, only the pH and SWC among these soil factors of diseased and killed *P. koraiensis* were higher than healthy P. *koraiensis* (Supplementary Figure 2).

### 3.3 The relative abundance of microbial and nematode taxa

**Figure 4:**
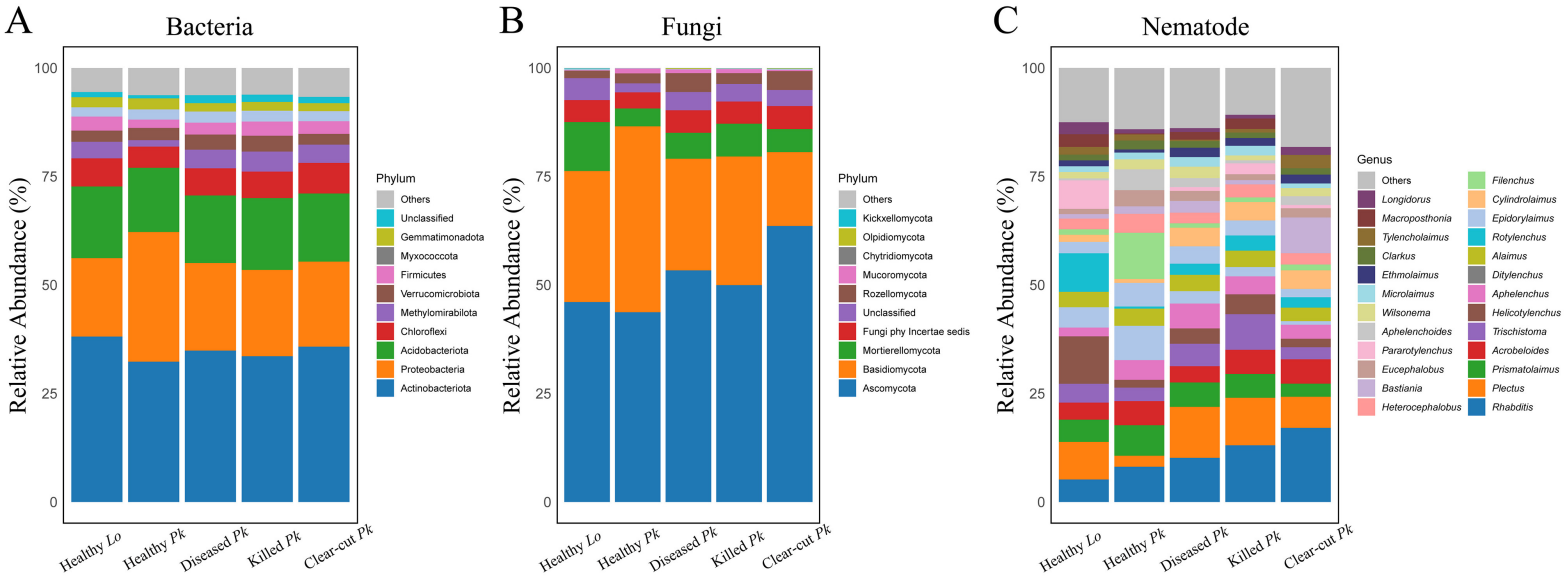
Stacked bars showing the relative abundances of bacterial **(A)**, fungal **(B)**, and nematode **(C)** communities at the phylum or genus level in soils collected from healthy larch *L. olgensis* stand resistant to PWD, healthy *P. koraiensis* stands, *P. koraiensis* stands diseased by PWD, and *P. koraiensis* stands killed by PWD as well as clear-cut *P. koraiensis* stands. The 10 most abundant bacterial and fungal phyla, as well as 25 most abundant nematode genera in soil were shown and the abundance of each taxon was averaged among replicated samples (*n* = 8).

The relative abundances of Proteobacteria and Gemmatimonadota was lower while the abundance of Methylomirabilota was higher in soil of diseased or killed *P. koraiensis* than healthy pines (Figure 4A and Supplementary Figure 3A). At genus level, the relative abundance of *Mycobacterium* was higher, while the relative abundance of *Microlunatus* and *Vicinamibacteraceae* were lower in soil of healthy *P. koraiensis* than diseased or dead *P. koraiensis* (Supplementary Figure 3D). The relative abundance of phylum Basidiomycota was lower but the abundance of Mortierellomycota was higher in soil of diseased or killed *P. koraiensis* than in soil of healthy pines (Figure 4B and Supplementary Figure 3B). At genus level, the relative abundance of *Solicoccozyma* was higher but that of *Sebacina* and *Cryptococcus* was lower in soil of diseased or killed *P. koraiensis* than in soil of healthy pines (Supplementary Figure 3E). We also found a lower relative abundance of genus *Aphelenchoides* in soil of killed *P. koraiensis* than in soil of healthy pines.

In addition, the relative abundances of Methylomirabilota, Chloroflexi, and Firmicutes in bacterial community were also lower in soil of healthy *P. koraiensis* than in soil of larch (Figure 4A and Supplementary Figure 3A). We also observed lower relative abundances of fungal genera *Mortierella, Trichocladium*, and *Penicillium* in the soil of healthy *P. koraiensis* than in soil of larch (Supplementary Figure 3E). In nematode community, the relative abundance of *Pararotylenchus* was lower but abundance of *Aphelenchoides* was higher in soil of healthy *P. koraiensis* than in soil of larch (Figure 4C and Supplementary Figure 3C).

### 3.4 Functional composition of microbial and nematode community

**Figure 5:**
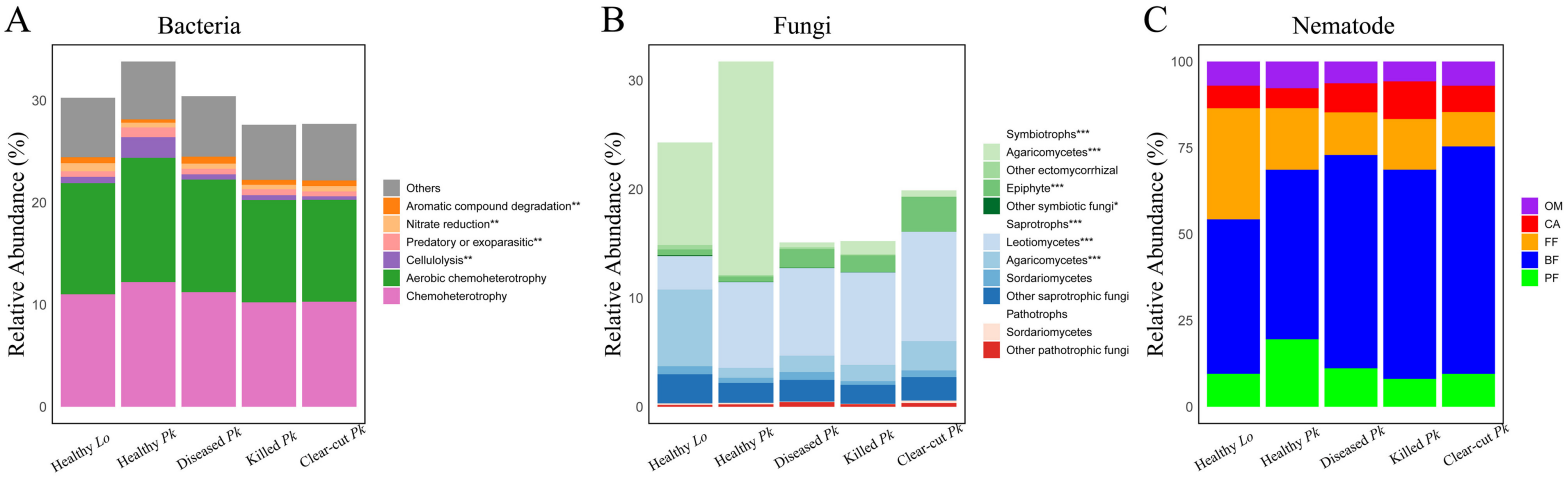
Stacked bars showing the relative abundances of the main bacterial ecological functions **(A)**, fungal guilds **(B)**, and nematode feeding groups **(C)** in soils collected from healthy larch *L. olgensis* stand resistant to PWD, healthy *P. koraiensis* stands, *P. koraiensis* stands diseased by PWD, and *P. koraiensis* stands killed by PWD as well as clear-cut *P. koraiensis* stands. Bacterial ecological functions with relative abundances <0.5% of total ASVs across all samples were grouped into “Others”. Abbreviations: PF, plant feeders; BF, bacterial feeders; FF, fungal feeders; CA, carnivores; OM, omnivores.

Bacterial ASVs related to functions in chemoheterotrophy and aerobic chemoheterotrophy had the highest relative abundance across all soil samples (Figure 5A). We found a lower abundance of bacterial ASVs of aromatic compound degradation but higher abundances of cellulolytic, predatory or exoparasitic bacterial taxa in the soil of healthy *P. koraiensis* than in soil of diseased or killed *P. koraiensis* (Figure 5A and Supplementary Figure 4A). In addition, we observed a lower relative abundance of bacteria related to nitrate reduction and aromatic compound degradation in soil of healthy *P. koraiensis* than in soil of larch (Figure 5A and Supplementary Figure 4A). The relative abundance of symbiotrophs in the fungal community, e.g., ectomycorrhizal fungi (EcM) dominated by Agaricomycetes, was significantly higher in soil of healthy *P. koraiensis* than in soil of diseased or killed *P. koraiensis* (Figure 5B and Supplementary Figure 4B). On the contrary, the relative abundance of saprotrophs was lower in the soil of healthy *P. koraiensis* than in the soil of larch (Figure 5B and Supplementary Figure 4B). Regarding the nematode community, the relative abundances of plant feeders seemed to be lower in the soil of diseased *P. koraiensis* than in the soil of healthy *P. koraiensis* (Figure 5C).

### 3.5 Co-occurrence network in the soil communities

**Figure 6:**
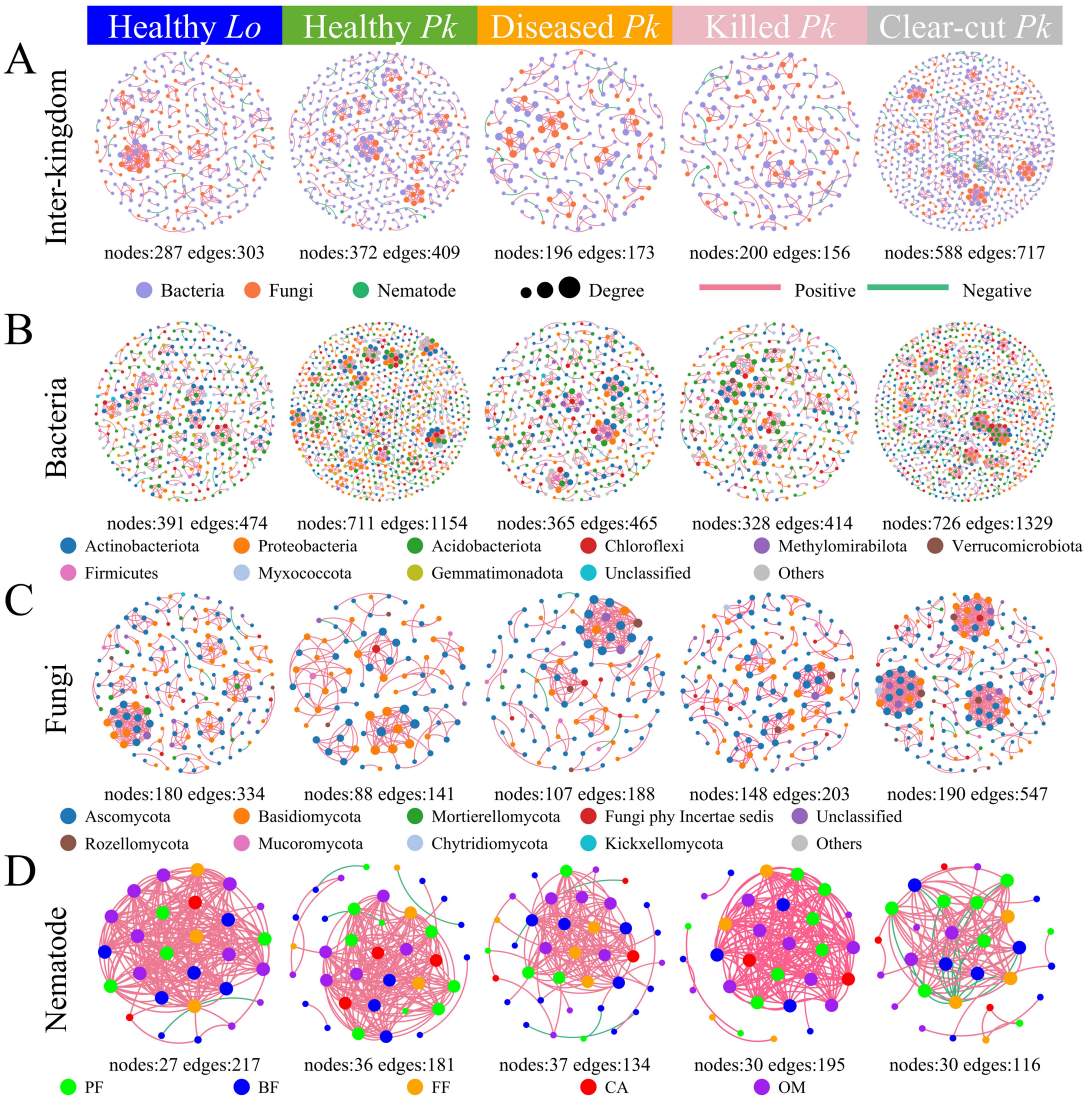
The co-occurrence networks within and between the taxa in the bacterial, fungal, and nematode communities in soils collected from healthy larch *L. olgensis* stand resistant to PWD, healthy *P. koraiensis* stands, *P. koraiensis* stands diseased by PWD, and *P. koraiensis* stands killed by PWD as well as clear-cut *P. koraiensis* stands. **(A)** In inter-kingdom co-occurrence networks among bacterial, fungal and nematode communities, purple nodes represent bacterial taxa, orange nodes represent fungal taxa, and green nodes represent nematode taxa. **(B)** In bacterial networks, nodes are colored for top 10 phyla. **(C)** In fungal networks, nodes are colored for top 10 phyla. **(D)** In the networks of nematodes, nodes are colored for 5 trophic groups due to the large number of genera. The nodes represented unique microbial ASVs or nematode genera, and the size of nodes was proportional to degree. A red link indicates a positive correlation and a green link negative correlation between the taxa. The absolute value of correlation >0.6 and *p*-value <0.05.

The numbers of nodes and edges in the inter-kingdom, bacterial, and fungal community networks were overall lower in soil of forest stands compared to those in soil of clear-cut field (Figures 6A–C). The inter-kingdom network complexity decreased following the progression of PWD, with the proportion of bacterial nodes decreasing but the proportion of fungal nodes increasing as the severity of PWD intensified (Figure 6A and Supplementary Table 3). The numbers of nodes and edges in bacterial community networks decreased but those numbers in fungal community networks increased over the progression of PWD (Figures 6B, C). The number of edges in nematode community networks was lower in soil of diseased *P. koraiensis* than in soil of healthy *P. koraiensis*, but this difference disappeared when trees were killed over time (Figure 6D). The average degree and density of the nematode community network first decreased in diseased pines but surpassed the original values in healthy pines at the later stage when the trees were killed (Supplementary Table 4).

## 4 Discussion

In the current study, we showed that the soil microbial community under the canopy of healthy pine stand was distinct from those under diseased or killed stands by PWD, but the communities of latter two stands did not differ. In contrast, we found similar microbial community composition in soil of the diseased or killed pines compared to the clear-cutting field, suggesting abolishment of the intimate interactions between host pines and the associated soil microbes due to PWD. Soil nematode community was less impacted, but its associations with bacterial community seemed simplified by the PWD, leading to decoupled connections among taxa in soil communities.

Many earlier studies have demonstrated an overall decline in soil bacterial diversity under the canopy of pines following the occurrence of PWD, and this is often attributed to the reduction in soil carbon or nutrient available to the diverse and abundant soil microbes (e.g., Shi et al., 2024; Guo et al., 2023). However, our results only revealed a transient increase in bacterial diversity in soil of diseased pines, and this increase diminished as the disease progressed when the pines were killed. This change might be associated to the incurred dynamics in plant or soil properties such as a higher soil pH by the disease as observed in our study, given that soil bacteria are highly sensitive to the changes in soil pH (Nicol et al., 2008; Rousk et al., 2010). On the other hand, it is noted that in our study the soils were collected from different stands at a distance of at least 5 km and the differences in these soil properties may not necessarily originate from the occurrence or development of PWD, but from spatial heterogeneity these soils have experienced. Considering that early studies mostly focused on *P. massoniana* that distributes in (sub)tropical region of China and has been shown distinct responses to PWD from the temperate species *P. koraiensis* used in our study (Ma et al., 2020). Although soil fungal community did not differ between diseased and healthy *P. koraiensis*, it exhibited a higher species richness in soil of killed *P. koraiensis* compared to the healthy pines. The increase is possibly a result of enrichment of necrotrophic fungi that were recruited to the rhizoplane of these fully destructed pines of deprived viability (Vicente et al., 2021). This assumption was supported by the enhanced proportion of saprotrophic fungi in soil of killed pines than in soil of healthy pines in our study. Together, these results showed that soil microbial community associated to host pines following PWD are possibly dependent not only on the pine species examined but also on the development stage of the PWD (Proença et al., 2017; Guo et al., 2020; Qu et al., 2020). However, it is noteworthy that our samplings encounter pseudoreplication issues due to the limited number of replicated pine stands within a developmental stage of PWD, which may overestimate the temporal influences of PWD highlighted in this study. In this sense, the results may not be fully extrapolated to other relevant studies, and the generalization of our findings needs sampling efforts with true replications.

We notice that the relative abundance of Proteobacteria in the bacterial community was lower in the soil of diseased or killed *P. koraiensis* following PWD than the healthy *P. koraiensis*. Such pattern was also observed in our previous study in which we compared microbial communities in multiple plant-soil compartments of *P. koraiensis* following PWD (Hou et al., 2025). Proteobacteria are common pathogens of many plant diseases (Li D. et al., 2024), thus its lower abundance in soil of diseased *P. koraiensis* may suggest strengthened immune system of this pine species induced by PWD (Wang et al., 2024). Similarly, we found a lower relative abundance of Basidiomycota, e.g. genus *Cryptococcus* in soil fungal community of the diseased and killed pines than healthy ones. Furthermore, a lower relative abundance of the fungal taxa *Penicillium* in soil of healthy *P. koraiensis* compared to in soil of PWD-resistant larch suggests a potential antagonistic role of this genus against nematodes (Sikandar et al., 2020; Nguyen et al., 2021; Hao et al., 2022), though further studies are needed to verify such roles.

Some studies show that the induced changes in the proportion of symbionts associated with pine roots by PWD may also contribute to the susceptibility of many pine species to the disease (Vicente et al., 2021; Chu et al., 2021; Jiao et al., 2023). In line with these studies, we indeed observed a lower proportion of symbiotroph, particularly the proportion of ectomycorrhizae Agaricomycetes in the soil of diseased or killed *P. koraiensis* than the healthy *P. koraiensis* and *L. olgensis*. This result is consistent with observations from another study using *P. massoniana* as host pines of PWD (Guo et al., 2023). The possible reason for the reduction of these mycorrhizal fungi is that PWD reduces the viability of the host pines leading to dead cells in the roots that do not transfer carbon to the EcM (Jiao et al., 2023). On the other hand, the reduced viability of host pines by PWD may provide opportunity for the saprotrophs that are readily decomposing the residuals of those pines destined to die. This assumption was indeed supported by the increased relative abundance of saprotrophs over the progression of PWD in the current study (Sapsford et al., 2022; Guo et al., 2023).

Overall, we did not observe changes in the diversity of soil nematode communities of *P. koraiensis* over the progression of PWD. This suggests that nematode communities in soil of pine forest may not be as sensitive to the disturbances like PWD as the microbial communities, likely due to their dependence on dynamics of microbial populations in the soil micro-food web (Wilschut and Geisen, 2021; Ji et al., 2023). Instead, we only found changes in relative abundance of several specific nematode genera following the PWD occurrence. For example, the abundance of fungal-feeding *Aphelenchoides* was reduced in soil of killed *P. koraiensis* than in healthy pines. This contrasts with the result of enriched saprotrophic fungi in the soil of the diseased pines because the spores and hyphae of these fungi are often considered food sources of these nematodes, suggesting a possibly decoupled trophic network in soil community resulting from PWD (Ruess et al., 2000). This possibility was further verified by the simplified inter- and within-kingdom networks of soil microbiota of the diseased and killed pines. Overall, we observed a decreased complexity (reduced numbers of nodes and edges) of the inter-kingdom networks in soil of *P. koraiensis* over the progression of PWD, suggesting a destruction of soil biotic interactions due to this disease. We specifically noticed that the numbers of nodes and edges in the bacterial network decreased and the numbers of fungal network tended to increase over the progression of PWD, suggesting a shift from bacterial-dominated to fungal-dominated networks in the soil microbial community as the PWD develops (Jiao et al., 2021; Ding et al., 2024). As network stability of a soil community was often strongly correlated with its functioning (Yuan et al., 2021), the decrease in the network complexity as a result of PWD may directly indicate mitigation in some soil functions. However, such speculation awaits verification that investigates biotic interactions among soil organisms and associated changes in soil functions (Delgado-Baquerizo et al., 2020).

## 5 Conclusion

Our study records the structural dynamics of soil microbial and nematode communities under the canopy of *P. koraiensis* over the progression of PWD. We conclude that both soil microbial and nematode communities exhibited compositional shifts over the time course of PWD, but the microbial community appears more sensitive to the disease development than the nematode community. We particularly show that the complexity of microbial networks as well as the inter-kingdom networks in the microbial food webs of *P. koraiensis* became simplified over the development of PWD, suggesting decoupled biotic interactions in the soil and potential mitigation of ecosystem functioning of pine forests following the disease. The findings of this study provide insights into the impacts of PWD on soil community structure, thus providing an important reference for better assessing the ecological consequences of this devastating disease.

## Data Availability

The data presented in this study are publicly available. The data can be found here: https://www.ncbi.nlm.nih.gov, accession PRJNA1356686.
