# Supplementary material for: Shifts in soil microbial and nematode communities over progression of pine wilt disease occurring in Pinus koraiensis stands
Source: Front Microbiol. 2025 Nov 24;16:1634289. doi: 10.3389/fmicb.2025.1634289 (PMC12682898; doi:10.3389/fmicb.2025.1634289)
Supplement: Supplementary file 1 [file Data_Sheet_1.pdf]

Supplementary Material

1 Supplementary Figures and Tables

1.1 Supplementary Tables

Supplementary Table 1. Pairwise PERMANOVA (permutation multivariate analysis of variance) results showing differences in bacterial, fungal, and nematode communities between soil origins. R^2 values (proportion of community variance explained) and Holm-adjusted *p*-values are shown. Abbreviations for soil origins: C, clear-cut *Pinus koraiensis*; H, healthy *P. koraiensis*; D, diseased *P. koraiensis*; K, killed *P. koraiensis*; L, healthy larch species (*Larix olgensis*). Bold values indicate significance at an adjusted *p*-value <0.05.

pairs	Bacteria (R^2, adjusted *p*-values)	Fungi (R^2, adjusted *p*-values)	Nematode (R^2, adjusted *p*-values)
C vs H	**0.76, 0.01**	**0.41, 0.01**	**0.29, 0.01**
C vs D	**0.31, 0.01**	**0.19, 0.01**	**0.15, 0.046**
C vs K	**0.41, 0.01**	**0.22, 0.01**	**0.18, 0.027**
C vs L	**0.52, 0.01**	**0.55, 0.01**	**0.31, 0.01**
H vs D	**0.79, 0.01**	**0.42, 0.01**	**0.29, 0.015**
H vs K	**0.81, 0.01**	**0.42, 0.01**	**0.34, 0.012**
H vs L	**0.74, 0.01**	**0.46, 0.01**	**0.30, 0.01**
D vs K	0.05, 0.509	0.07, 0.32	0.04, 0.842
D vs L	**0.57, 0.01**	**0.60, 0.01**	**0.25, 0.01**
K vs L	**0.62, 0.01**	**0.59, 0.01**	**0.23, 0.02**

Supplementary Table 2. Coefficient of determination (R^2) of the effects of soil physicochemical properties on bacteria, fungi, and nematode communities in CCA (Canonical Correspondence Analysis). Abbreviations: SWC, soil water content; TC, total carbon; TN, total nitrogen; TP, total phosphorus. Bold values indicate significance at an adjusted *p*-value <0.05.

Soil physicochemical properties	Bacteria (R^2, *p*-value)	Fungi (R^2, *p*-value)	Nematode (R^2, *p*-value)
pH	**0.94, < 0.001**	**0.98, < 0.001**	**0.68, < 0.001**
SWC	**0.49, < 0.001**	**0.54, < 0.001**	**0.54, < 0.001**
TC	**0.31, 0.003**	**0.60, < 0.001**	0.11, 0.110
TN	**0.52, < 0.001**	**0.80, < 0.001**	**0.19, 0.018**
TP	**0.47, < 0.001**	**0.78, < 0.001**	**0.45, < 0.001**

Supplementary Table 3. Topological properties of inter-kingdom networks across soil origins.

Topological properties		Healthy *Lo*	Healthy *Pk*	Diseased *Pk*	Killed *Pk*	Clear-cut *Pk*
		Soil origins				
Nodes	All	287	372	196	200	588
	Bacteria	206 (71.78%)	317 (85.22%)	152 (77.55%)	146 (73.00%)	465 (79.08%)
	Fungi	80 (27.87%)	52 (13.98%)	43 (21.94%)	51 (25.50%)	119 (20.24%)
	Nematode	1 (0.35%)	3 (0.81%)	1 (0.51%)	3 (1.50%)	4 (0.68%)
Edges	All	303	409	173	156	717
	Bacteria-Bacteria	131 (43.23%)	263 (64.30%)	86 (49.71%)	87 (55.77%)	421 (58.72%)
	Bacteria-Fungi	121 (39.93%)	113 (27.63%)	63 (36.42%)	52 (33.33%)	225 (31.38%)
	Fungi-Fungi	50 (16.50%)	29 (7.09%)	23 (13.29%)	14 (8.97%)	66 (9.21%)
	Bacteria-Nematode	1 (0.33%)	4 (0.98%)	1 (0.58%)	3 (1.92%)	4 (0.56%)
	Fungi-Nematode	1 (1.08%)	0	0	0	0
	Nematode-Nematode	0	0	0	0	0
Positive correlation		278 (91.75%)	278 (91.75%)	156 (90.17%)	144 (92.31%)	636 (88.70%)
Negative correlation		25 (8.25%)	25 (8.25%)	17 (9.83%)	12 (7.69%)	81 (11.30%)
Average degree		2.111	2.111	1.765	1.560	2.439
Density		0.007	0.007	0.009	0.008	0.004
Modularity		0.916	0.916	0.966	0.978	0.974

Supplementary Table 4. Topological properties of single-community networks across soil origins.

Topological properties		Soil origins: Healthy *Lo*	Healthy *Pk*	Diseased *Pk*	Killed *Pk*	Clear-cut *Pk*
Bacteria	Positive correlation	454 (95.78%)	1079 (93.50%)	445 (95.70%)	402 (97.10%)	1259 (94.73%)
	Negative correlation	20 (4.22%)	75 (6.50%)	20 (4.30%)	12 (2.90%)	70 (5.27%)
	Average	2.425	3.246	2.548	2.524	3.661
	Density	0.006	0.005	0.007	0.008	0.005
	Modularity	0.972	0.974	0.965	0.964	0.947
Fungi	Positive correlation	329 (98.50%)	139 (98.58%)	185 (98.40%)	203 (100%)	540 (98.72%)
	Negative correlation	5 (1.50%)	2 (1.42%)	3 (1.60%)	0	7 (1.28%)
	Average	3.711	3.205	3.514	2.743	5.758
	Density	0.021	0.037	0.033	0.019	0.030
	Modularity	0.876	0.883	0.736	0.928	0.803
Nematode	Positive correlation	216 (99.54%)	178 (98.34%)	131 (97.76%)	195 (100%)	102 (87.93%)
	Negative correlation	1 (0.46%)	3 (1.66%)	3 (2.24%)	0	14 (12.07%)
	Average	16.074	10.056	7.243	13.000	7.733
	Density	0.618	0.287	0.201	0.448	0.267
	Modularity	0.063	0.107	0.196	0.050	0.179

1.2 Supplementary Figures

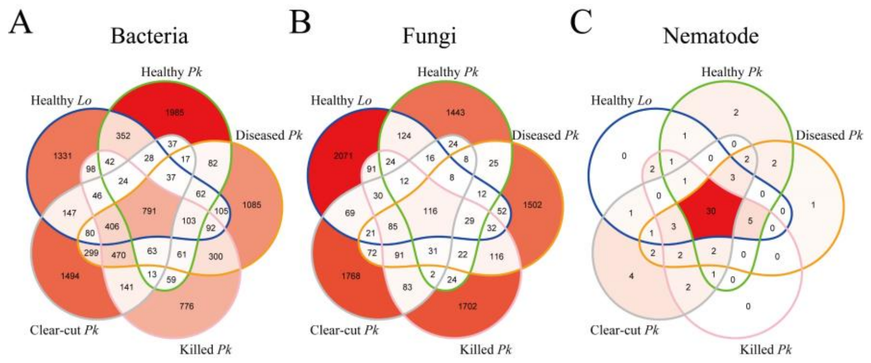

Supplementary Figure 1. Venn diagram of bacterial (A), fungal (B), and nematode (C) communities (bacteria and fungi at the ASV level, nematode at the genus level).

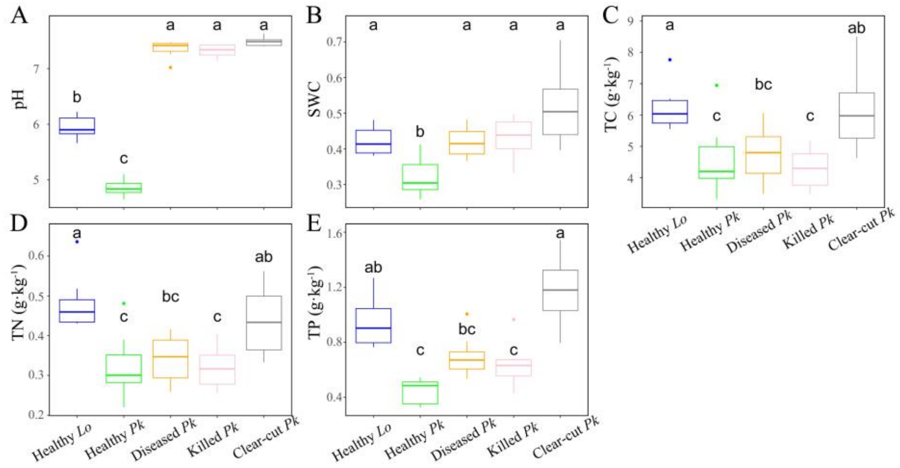

Supplementary Figure 2. Soil physicochemical properties at different soil origins. Different letters represent significant differences. Soil variables are as follows: SWC, soil water content; TC, total carbon; TN, total nitrogen; TP, total phosphorus.

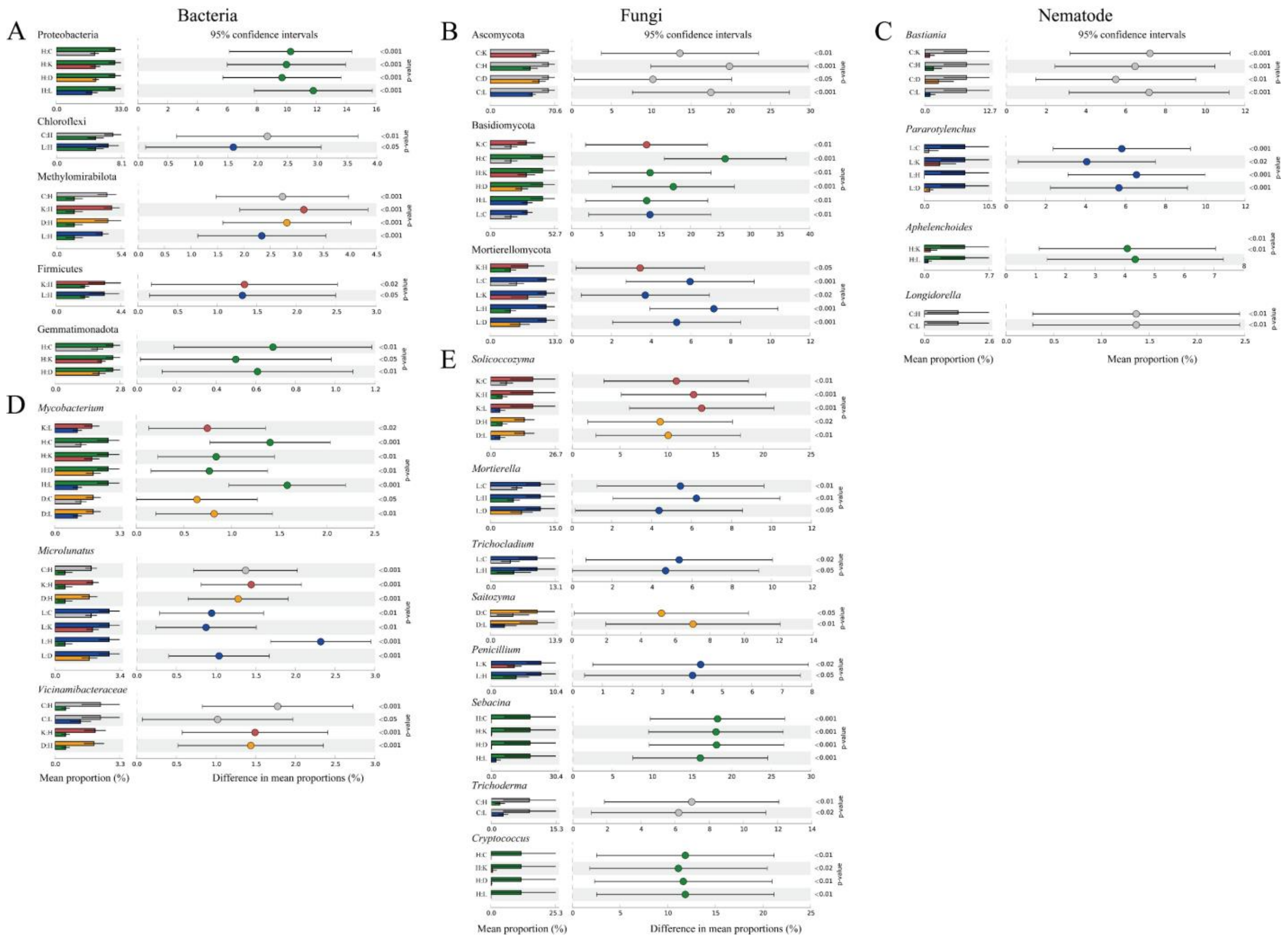

Supplementary Figure 3. Differential abundance analysis of bacterial and fungal communities at the phylum (A, B) and genus (D, E) levels and nematode community at the genus level (C) between soil origins was performed using the Kruskal-Wallis test with Benjamini-Hochberg correction in STAMP (Statistical analysis of taxonomic and functional profiles) software. Abbreviations for soil origins: C, clear-cut *P. koraiensis*; H, healthy *P. koraiensis*; D, diseased *P. koraiensis*; K, killed *P. koraiensis*; L, healthy larch species (*L. olgensis*).

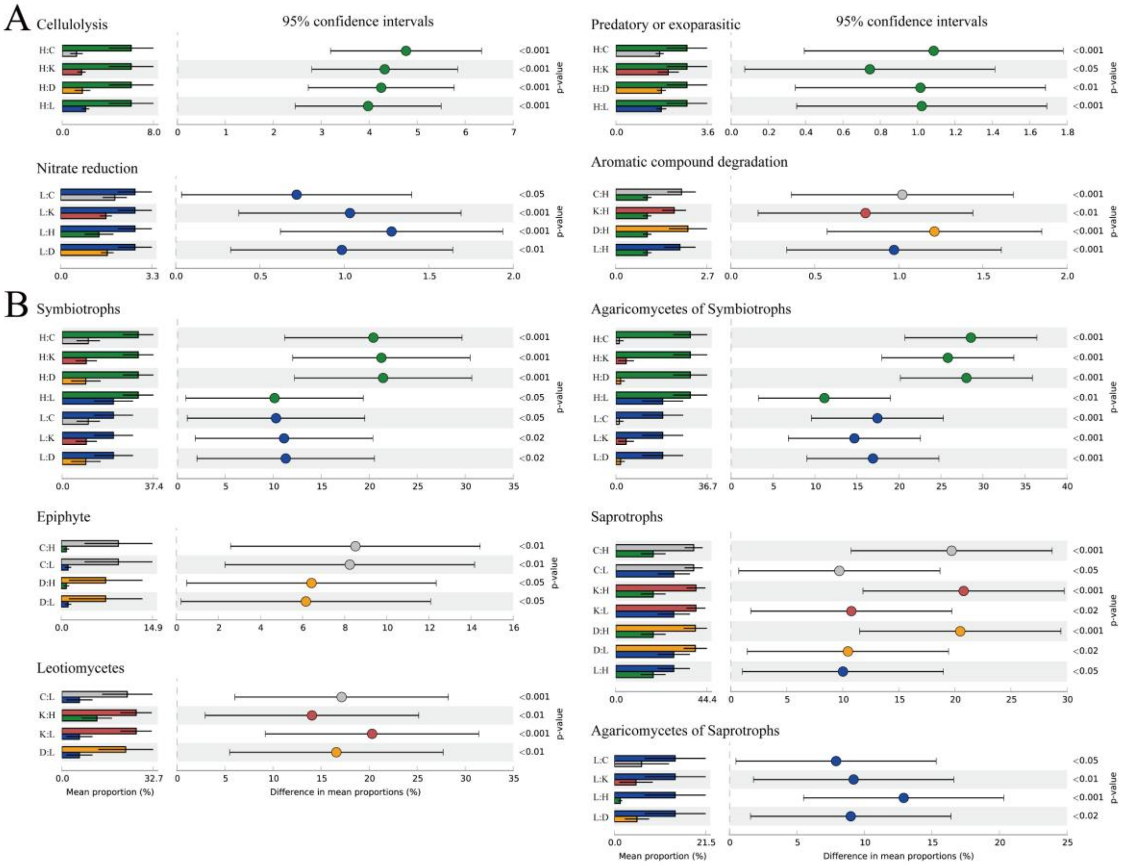

Supplementary Figure 4. Results from STAMP (Kruskal-Wallis test with Benjamini-Hochberg correction) reveal significant differences of main bacterial ecological functions (A) and fungal guilds (B) among soil origins associated with the progression of PWD in *P. koraiensis* or in healthy larch. Bacterial ecological functions with relative abundances <0.5% of total sequences were grouped into "Others". Abbreviations for soil origins: C, clear-cut *P. koraiensis* stands; H, healthy *P. koraiensis* stands; D, *P. koraiensis* stands diseased by PWD; K, *P. koraiensis* stands killed by PWD; L, healthy *L. olgensis* stands resistant to PWD.
